# *Filaggrin* gene polymorphisms are associated with atopic dermatitis in women but not in men in the Caucasian population of Central Russia

**DOI:** 10.1371/journal.pone.0261026

**Published:** 2021-12-09

**Authors:** Volodymyr Dvornyk, Irina Ponomarenko, Tatyana Belyaeva, Evgeny Reshetnikov, Mikhail Churnosov

**Affiliations:** 1 Department of Life Sciences, College of Science and General Studies, Alfaisal University, Riyadh, Saudi Arabia; 2 Department of Medical Biological Disciplines, Belgorod State University, Belgorod, Russia; Seoul National University College of Medicine, REPUBLIC OF KOREA

## Abstract

**Background and purpose:**

This study aimed to analyze the gender-specific association of the *filaggrin* (*FLG*) gene polymorphisms with atopic dermatitis (AD) in Caucasians from the central region of Russia.

**Methods:**

The study sample consisted of 906 female (including 474 patients with AD and 432 controls) and 406 male (such as 226 patients with AD and 180 controls) participants. Genotyping of ten polymorphisms of the *FLG* gene was done. The logistic regression was used to analyze the associations. A total of 125 SNPs (seven AD-associated SNPs and 118 proxy SNPs, r^2^≥0.8) *FLG* gene were used for the *in silico* functional annotation analysis in the females.

**Results:**

Significant associations were identified between seven SNPs of the *FLG* gene (rs12130219, rs61816761, rs558269137, rs12144049, rs3126085, rs471144, rs6661961) and AD in females: rs12144049 was associated independent individually (for allele C OR = 1.71, 95%Сl 1.19–2.46, р_perm_ = 0.004 and OR = 1.76, 95%Сl 1.18–2.63, р_perm_ = 0.006 according to the additive and dominant genetic models, respectively) and seven SNPs of the *FLG* gene within 14 haplotypes. Haplotype GGT [rs61816761-rs3126085-rs12144049] showed the strongest association (OR = 0.55, р_perm_ = 0.001). No association between the analyzed SNPs and AD was determined in the male group. The subsequent bioinformatic analysis predicted the SNPs of the *FLG* gene that possessed epigenetic and non-synonymous effects, were involved in the control of gene expression and alternative splicing of genes that contribute to AD pathophysiology.

**Conclusion:**

Polymorphisms of the *FLG* gene are associated with AD in females but not in males in the Caucasian population of Central Russia.

## Introduction

Atopic dermatitis (AD), also called eczema, is a chronic, relapsing, pruritic, inflammatory skin disease [1]. The prevalence of AD is high, up to 16% in the general practice population (lifetime ever diagnosed) with about 20% of the affected individuals having moderate to severe symptoms [2]. AD is characterized by chronic lichenified or excoriated plaques occurring in typical areas, such as the head, neck, flexural areas, and extensive itching lesions [3]. Patients with AD are more likely to have allergic rhinitis and asthma [4]; they frequently suffer from concomitant depression, anxiety, and attention deficit hyperactivity disorder [5,6]. AD may also be a risk factor for schizophrenia, eating disorder, and obsessive-compulsive disorder [6]. AD significantly decreases the quality of life of patients and their families, affecting physical and psychological well-being, social functioning, and economic costs [5].

AD has a higher incidence among females than males [2,4,7]. In particular [8], documented the higher prevalence of eczema during adolescence in females and the two-fold predominance throughout the reproductive period in a population sample of 266,733 from Scotland. Similar results were reported for atopic eczema in a sample of about 30,000 subjects from Great Britain: individuals with adult-onset atopic eczema are more likely to be women (OR = 1.66–1.75) [9]. In a systematic review and meta-analysis of risk factors associated with AD [10], reported a pooled OR for gender (male) equal to 0.67, thus suggesting a higher prevalence of the disease in females.

Profound alterations in skin barrier function and immunologic abnormalities are considered the key factors affecting the development and severity of AD [1]. The etiology of AD is multifactorial involving interactions between genetics, immune, and environmental factors [10,11]. Familial and twin studies have demonstrated that AD is a highly heritable disease: genetic factors account for nearly 90% of the disease susceptibility variance [12,13].

The filaggrin (*FLG*) gene is one of the most significant known risk factors for AD [11]. The gene is located on chromosome 1q21.3 in a region of the epidermal differentiation complex and encodes the structural protein, which is a major element in the stratum corneum [14]. FLG plays a key role in the development of the epidermis and the skin barrier [15]. FLG is also broken down into organic acids, which maintain the pH gradient and antimicrobial activity of the epidermis [10]. A meta-analysis of 24 studies on *FLG* null mutations determined a 3-fold increased risk of AD in the carriers of one or more null variants [16]. More than 300 *FLG* loss-of-function variants were identified [11] and more than 20 of them were associated with susceptibility to AD [17]. Several genome-wide association studies (GWAS) identified more than ten additional susceptibility loci of the *FLG* gene for AD [18–23].

However, despite the ample evidence about the *FLG* gene association with AD (see [11] for review) and a role of the female gender as a non-modifiable risk factor for the disease [8–10], the studies of the gender-specific relationships between the *FLG* gene and risk for AD are limited [24,25]. We hypothesized that gender might influence the association between *FLG* gene polymorphisms and AD. The present study was aimed to analyze gender-specific associations of the *FLG* gene with AD in Caucasians from the central region of Russia.

## Materials and methods

### Study subjects

All participants of the study provided informed consent before enrolment. The study protocol was approved by the Ethical Review Committee of Belgorod State University. In total, 906 females (including 474 patients with AD and 432 controls) and 406 males (such as 226 patients with AD and 180 controls) of Russian origin, born and living in the central region of Russia [26,27] were recruited during the 2010–2018 period through dermatovenerological dispensaries at Belgorod and Kursk Regions. The diagnosis of AD was verified by qualified dermatologists. Patients were diagnosed with AD according to the UK Diagnostic Criteria [28]. AD severity was assessed using the Eczema Area and Severity Index (EASI) [29]. All controls were clinically assessed to have no AD, other skin and atopic diseases (asthma, hay fever, allergic conjunctivitis, sensitization to allergens (air pollutants, food, medication, domestic animals, indoor allergens, etc.)), a family history of atopic diseases [30]. The cases and control group without any severe chronic disorders [31]. Baseline and clinical characteristics of the case and control groups are shown in Table 1. Among females and males, the control groups were matched to the AD patients by age, body mass index, and the other characteristics (p>0.05).

**Table 1:** Phenotypic and clinical characteristics of the study participants.

Parameters	Females (n = 906)			Males (n = 406)		
	Controls, mean ± SD, % (n)	AD patients, mean ± SD, % (n)	p	Controls, mean ± SD, % (n)	AD patients, mean ± SD, % (n)	p
N	432	474	-	180	226	-
Age, years (min–max)	41.14±15.02 (19–88)	40.52±15.31 (19–85)	0.32	45.88±15.42 (18–87)	46.37±17.31 (19–86)	0.28
BMI, kg/m2	25.12±5.13	25.01±5.07	0.66	24.03±5.04	24.25±5.30	0.45
Region of residence (age of onset), urban/rural area	78.94/21.06 (341/91)	74.99/25.11 (355/119)	0.17	83.33/16.67 (150/30)	80.09/19.91 (181/45)	0.48
Current smoking	6.71 (29)	7.17 (34)	0.89	24.44 (44)	25.66 (58)	0.87
Alcohol consumption	25.92 (112)	25.53 (121)	0.95	60.55 (109)	64.60 (146)	0.46
Social class*:			0.21			0.78
I/II	44.21 (191)	48.31 (229)		41.11 (74)	42.04 (95)	
III	45.84 (198)	43.67 (207)		46.67 (84)	46.02 (104)	
IV/V	9.95 (43)	8.02 (38)		12.22 (22)	11.94 (27)	
Allergic disorders (asthma, hay fever, allergic conjunctivitis, sensitization to allergens)	-	62.02 (294)	**-**	-	50.88 (115)	-
Family history of allergic diseases (AD, asthma, hay fever)	-	39.87 (189)	-	-	34.96 (79)	-
Age of self-reported AD onset, years	-	36.24±17.52	-	-	43.55±17.31	-
AD severity (identified by EASI):	-		-	-		-
Mild		55.28 (262)			59.73 (135)	
Moderate		39.66 (188)			36.73 (83)	
Severe		5.06 (24)			3.54 (8)	

* Registrar General’s social class: *I*, professional; *II*, managerial and technical; *III*, skilled; *IV*, partly skilled; *V*, unskilled.

### SNPs selection

Ten common SNPs of the *FLG* gene (rs61816761, rs12130219, rs3126085, rs558269137, rs6661961, rs10888499, rs471144, rs4363385, rs77199844, and rs12144049) were selected for the study based on the following criteria [32,33]: previously reported associations with AD (eczema) and functional relevance. The functionality of the selected loci was examined using the HaploReg database (*in silico* analysis) [34].

All ten selected loci were AD-associated according to the previous reports (nine SNPs were GWAS-significant) (S1 Table) and had regulatory significance (S2 Table). Also, five loci were previously showed association with some allergic disorders of the skin (psoriasis, ichthyosis vulgaris) and other organs (hay fever, asthma, etc.) (S1 Table).

### Genotyping

Genomic DNA was isolated from 4–5 ml of the peripheral blood samples using the phenol/chloroform extraction technique (as described earlier [35,36].

SNP genotyping was performed using the MALDI-TOF mass spectrometry iPLEX platform (Agena Bioscience Inc., San Diego, CA, USA). For the quality control, about 5% of the samples were selected randomly and subjected to the repeatability test [37,38] that yielded 100% reproducibility.

### Statistical analysis

The chi-squire test was applied to check the observed allele and genotype frequencies for correspondence to the Hardy–Weinberg equilibrium [39]. Logistic regression was used to analyze the association between the SNPs of the *FLG* gene and AD [40]. Age and BMI were applied as quantitative covariates. The adaptive permutation test was utilized to correct for multiple comparisons [41]. All the above computations were performed using the PLINK package [42]. The Bonferroni adjusted value P_perm_ ≤ 0.008 (0.05/6) was accepted as statistically significant given the numbers of the analyzed genetic models n = 3 [43], and the number of the groups compared (n = 2). The given sample sizes for females (474 patients with AD and 432 controls) and males (226 patients with AD and 180 controls) were sufficient to detect differences in allelic frequencies between the affected subjects and controls, respectively, at OR = 1.31–1.75 and OR = 1.49–2.25 for the additive model, OR = 1.53–1.79 and OR = 1.91–2.35 for the dominant model, OR = 1.56–19.0 and OR = 1.93–115.00 for the recessive model (at 80% power, ɑ = 0.05 for 2-sided test). Statistical power for each SNP was estimated using Quanto 1.2.4 [44]. The «Four gamete frequencies» algorithm of linkage disequilibrium with D’ > 0.80 realized in the Haploview software [45] was selected to infer haplotype blocks. For the haplotype association, value p_perm_ ≤ 0.025 was adopted as statistically significant (based on the numbers of groups compared n = 2).

### SNPs functionality effects

To estimate the potential downstream functional effects of the AD-associated variants and their proxies (r^2^≥0.8) [46,47], we used the available data of epigenetic effects (HaploReg [34]), non-synonymous functional predictions (SIFT and PolyPhen-2 databases [48,49]), expression and alternative splicing quantitative traits (GTExconsortium atlas, [50]). HaploReg and European population data of the 1000 Genomes Project Phase 1 were used to identify variants in close linkage disequilibrium (r^2^ ≥ 0.8) with the AD-associated variants [51,52].

## Results

### SNP association analyses

S3 Table shows the allele and genotype distribution of the studied SNPs in females and males. No departure from the Hardy–Weinberg equilibrium was observed in both studied groups (p>0.005 and p_bonf_>0.05). Variant allele C rs12144049 was found to be significantly associated with the increased AD risk in the additive (OR = 1.71, 95%Сl 1.19–2.46, р = 0.004, р_perm_ = 0.004, power—99.71%) and dominant (OR = 1.76, 95%Сl 1.18–2.63, р = 0.006, р_perm_ = 0.006, power—98.53%) genetic models only among females (Table 2). No statistically significant association between SNPs of the *FLG* gene and AD was observed in the male group (Table 2).

**Table 2:** Associations of the *FLG* gene polymorphisms with AD in females and males.

SNP	MAF	n	Additive model				Dominant model				Recessive model			
			OR	95%CI		Р	OR	95%CI		Р	OR	95%CI		Р
				L95	U95			L95	U95			L95	U95	
Females														
rs12130219	G	892	0.96	0.71	1.30	0.782	1.05	0.72	1.54	0.792	0.61	0.27	1.34	0.216
rs558269137	delACTG	892	2.02	0.80	5.04	0.135	2.02	0.80	5.04	0.135	-	-	-	-
rs6661961	T	896	0.99	0.76	1.27	0.917	0.92	0.62	0.98	0.665	1.09	0.68	1.75	0.734
rs3126085	A	886	1.60	1.07	2.38	0.021	1.74	1.10	2.77	0.019	1.86	0.55	6.27	0.318
rs12144049	C	862	**1.71**	**1.19**	**2.46**	**0.004**	**1.76**	**1.18**	**2.63**	**0.006**	2.82	0.75	10.64	0.127
rs61816761	A	898	4.59	0.53	39.59	0.166	4.59	0.53	39.59	0.166	-	-	-	-
rs471144	G	872	1.23	0.75	2.02	0.408	1.12	0.65	1.91	0.684	0.01	0.00	inf	0.999
rs10888499	C	896	0.84	0.62	1.14	0.271	0.68	0.47	1.00	0.047	1.69	0.76	3.75	0.197
rs77199844	delAT	884	0.83	0.46	1.50	0.532	0.83	0.46	1.50	0.532	-	-	-	-
rs4363385	T	872	0.95	0.73	1.24	0.691	0.81	0.54	1.22	0.313	1.13	0.70	1.82	0.624
Males														
rs12130219	G	398	1.35	0.84	2.17	0.217	1.56	0.88	2.75	0.128	0.96	0.28	3.27	0.952
rs558269137	delACTG	386	0.77	0.22	2.78	0.695	0.77	0.22	2.78	0.695	-	-	-	-
rs6661961	T	398	1.09	0.73	1.61	0.688	1.39	0.78	2.47	0.259	0.75	0.35	1.61	0.466
rs3126085	A	396	1.96	0.99	3.88	0.054	1.88	0.91	3.92	0.090	0.01	0.00	inf	0.999
rs12144049	C	382	1.13	0.68	1.86	0.645	1.42	0.76	2.65	0.275	0.49	0.13	1.80	0.282
rs61816761	A	396	0.01	0.00	inf	0.999	0.01	0.00	inf	0.999	-	-	-	-
rs471144	G	404	0.66	0.29	1.50	0.317	0.69	0.29	1.65	0.406	0.01	0.00	inf	0.999
rs10888499	C	398	0.78	0.51	1.22	0.276	0.73	0.42	1.29	0.280	0.74	0.26	2.05	0.557
rs77199844	delAT	402	1.54	0.67	3.54	0.309	1.54	0.67	3.54	0.309	-	-	-	-
rs4363385	T	392	1.05	0.70	1.55	0.821	1.05	0.58	1.89	0.871	1.09	0.52	2.27	0.828

All results were obtained after adjustment for covariates.

ОR: Odds ratio.

95%CI: 95% confidence interval.

P values ≤ 0.008 are shown in bold.

### Haplotype association analyses

**Fig 1:**
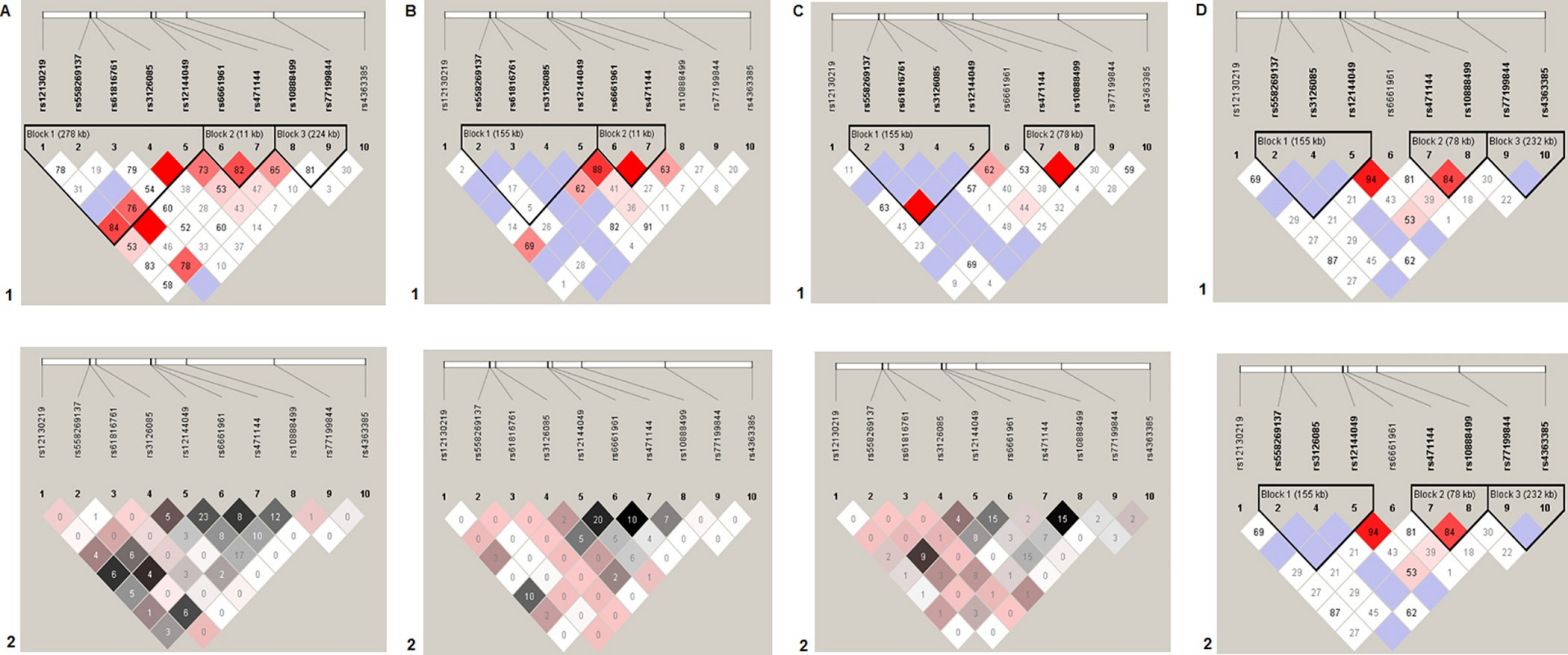
Linkage disequilibrium (LD) between ten studied SNPs of the *FLG* gene in females (A and B) and males (C and D). A and C—AD patients, B and D—control group. LD values are presented as Lewontin’s standardized coefficient D′ (Figure sections 1) and the square of the Pearson’s correlation coefficient (r^2^) (Figure sections 2) between the SNPs.

The LD of the *FLG* gene SNPs was analyzed separately in females and males. The haploblock structures were different between 1) AD patients and controls in both females and males and 2) males and females in both the patients and controls (Fig 1). The haplotypes manifested the association with the disease only in women but not men (Table 3). The strongest association was demonstrated by haplotype GGT [rs61816761-rs3126085-rs12144049] (OR = 0.55, p = 0.00006, р_perm_ = 0.001).

**Table 3:** Significant associations of the *FLG* gene haplotypes with AD in females (P_perm_ ≤ 0.025).

№	SNPs	Haplotypes	Frequency		OR	P	P_perm_
			Cases	Controls			
1	rs3126085|rs12144049	GC	0.232	0.154	1.73	0.003	0.009
2	rs3126085|rs12144049	GT	0.621	0.748	0.56	0.0001	0.001
3	rs61816761|rs3126085	GG	0.851	0.907	0.58	0.007	0.003
4	rs61816761|rs3126085	GA	0.149	0.093	1.06	0.021	0.016
5	rs558269137|rs61816761|rs3126085	WGG	0.822	0.894	0.55	0.002	0.001
6	rs3126085|rs12144049|rs6661961	GTT	0.124	0.195	0.56	0.003	0.017
7	rs61816761|rs3126085|rs12144049	GGT	0.632	0.751	0.55	0.00006	0.001
8	rs61816761|rs3126085|rs12144049	GGC	0.221	0.154	1.61	0.010	0.025
9	rs61816761|rs3126085|rs12144049|rs6661961	GGTT	0.124	0.194	0.55	0.003	0.016
10	rs558269137|rs61816761|rs3126085|rs12144049	WGGT	0.629	0.752	0.56	0.0001	0.001
11	rs12130219|rs558269137|rs61816761|rs3126085|rs12144049	AWGGT	0.425	0.544	0.61	0.0004	0.002
12	rs558269137|rs61816761|rs3126085|rs12144049|rs6661961	WGGTT	0.115	0.193	0.51	0.0009	0.005
13	rs12130219|rs558269137|rs61816761|rs3126085|rs12144049|rs6661961	AWGGTT	0.097	0.181	0.44	0.0002	0.001
14	rs12130219|rs558269137|rs61816761|rs3126085|rs12144049|rs6661961|rs471144	AWGGTTT	0.079	0.150	0.44	0.0007	0.004

All results were obtained after adjustment for covariates.

OR: Odds ratio.

P: Significance level.

P_perm_: Significance level after the adaptive permutation test (1000 permutations).

### Functional SNP predictions

#### Regulatory and non-synonymous effects

The results of the bioinformatic analysis of the genomic and epigenetic effects for the seven AD risk loci of the *FLG* gene and 118 proxy SNPs (r^2^≥0.8) in females are given in S4 Table. According to the HaploReg database, 30 SNPs were located in exons of the *FLG* gene. Among them, locus rs61816761 is a nonsense mutation (R501X) and rs558269137 is a frameshift variant (2282delACTG). More than 20 SNPs were in strong LD with rs3126085 (S5 Table). One SNP, rs201584430, linked to the risk SNP rs12130219, was located in the *FLG-AS1* gene splice donor site. Ten proxy SNPs were found in introns and 85 loci were located in the 3’- or/and 5’-UTR regions of seven genes (*FLG*, *FLG-AS1*, *FLG2*, *LCE5A*, *CRNN*, *RP1-91G5*.*3*, and *HRNR*) (S4 Table).

Most of the proxy SNPs have significant epigenetic effects. For example, rs201584430, which is in strong LD with the risk locus rs12130219, has a DNA position in the histone modification region corresponding to enhancer and promoter elements (24 and 6 tissues respectively), DNase hypersensitivity chromatin state region (4 tissues), and a genomic region with 25 transcription factors binding loci. Another proxy, rs17597997 (inked to the risk SNP rs6661961), was highly enriched for promoters (14 tissues), enhancers (18 tissues), and DNase hypersensitive (42 tissues) regions across multiple cell lines, tissues, and organs.

#### Expression and splicing QTLs

All seven AD risk SNPs were expression quantitative trait loci associated with transcription of 16 target genes (S6 Table); six risk SNPs had the skin-specific transcript associations with six genes (*CRNN*, *FLG*, *FLG2*, *FLG-AS1*, *LINGO4*, *RP1-91G5*.*3*) (S7 Table). The 100 proxy SNPs of the five AD risk loci affected mRNA transcript abundance of twelve genes (S8 Table), including six genes with the skin-specific expression (S9 Table).

The effects of the analyzed SNPs on the alternative splicing are shown in S10 Table. The rs6661961 locus individually and 21 SNPs linked to it and rs3126085 were the splicing quantitative trait loci for two genes (*RP11-107M16*.*2* and *CRNN*).

## Discussion

In the present study, we found that polymorphisms of the *FLG* gene are associated with AD in women but not in men in the Caucasian population of the central region of Russia. Locus rs12144049 was associated with the disease individually (OR = 1.71–1.76, р_perm_≤0.006) and seven SNPs were associated within 14 haplotypes. Importantly, the OR value for the risk allele C rs12144049 of the *FLG* gene determined in the present study (OR = 1.71–1.76) was similar to those previously reported by the GWAS of European populations: OR = 1.53 [22] and OR = 1.39 [23].

FLG is an important structural protein that is responsible for the keratinization, moisturization, and antimicrobial functions of the skin stratum corneum [15,53]. It is necessary for the generation of the natural moisturizing factor, which is produced upon FLG deamination and breakdown. The natural moisturizing factor is important for the maintenance of stratum corneum hydration and also reduces its pH to about 5.5 [54]. Epidermal insufficiency of FLG increases trans-epidermal water loss, causing the drying and cracking of the epidermis; FLG insufficiency also leads to aberrant keratinocyte differentiation, resulting in inadequate skin lipid content [55]. The insufficiency in the epidermal barrier results in the penetration of allergens and microorganisms [54]. Skin barrier defects have been considered an initial step in developing AD [53].

The key role of the null mutations (R501X, 2282del4, etc.) of the *FLG* gene in the epidermal barrier deficiency and AD was previously demonstrated [11,56,57]. Besides, several recent GWAS of AD suggested SNPs of the *FLG* gene as possible risk factors for the disease [18–23], which was supported by the results of the present study too. Importantly, both loss-of-function mutations and SNPs of the *FLG* gene are also a risk factor for other atopic conditions, e.g., asthma and hay fever thus suggesting that FLG deficiency may have a broader systemic significance [58–61]. For example, the rs61816761 variant of the *FLG* gene was 1.32-fold more common in patients suffering only from eczema when compared to those suffering only from hay fever and 1.26-fold more common as compared with asthma-only cases [58]. Likewise, variant rs12144049 was significantly associated with both AD [22,23]; present study) and asthma [59].

The present study showed the association of the *FLG* gene with AD only in women. There is a limited number of studies of gender-related differences in associations of candidate genes and AD [24,62] found no evidence of an interaction between *FLG* genotypes and sex in children aged 6 months to 11 years. On the other hand, there is evidence about the higher prevalence of AD among females at adolescence and adulthood [2,4,7–10] that may suggest a role of sex hormones on the expression of this allergic disease [8]. The female sex steroids, oestrogens and progesterone, may produce the immune stimulatory effects [63]. The reactivity to allergens increases in women during a mid-menstrual cycle that suggests important modulation of immune responses by sex steroid levels [64]. Oestrogen and testosterone produce opposite effects: pro-inflammatory and anti-inflammatory, respectively [63,65]. The effects of sex steroids may explain the significant sex differences and reversals observed in atopy (asthma, AD, etc.) [8] particularly the gender reversal in prevalence occurring at the time of hormonal changes. AD has a male predominance during childhood and female predominance after adolescence [4,8,10]. Apparently, during the reproductive years, particularly during puberty, higher levels of female sex hormones elevate an atopic predisposition in females, while male hormones may have a protective effect [8]. Given this, it is reasonable to suppose that sex hormones may modulate phenotypic effects of the *FLG* gene in the course of AD and determine the observed gender‐related differences in the associations of the *FLG* gene polymorphisms with AD.

The *in silico* analysis suggested relationships of the seven risk SNPs *FLG* gene and 118 proxy SNPs (r^2^≥0.8) with the skin-specific expression of several genes (*CRNN*, *FLG*, *FLG2*, *FLG-AS1*). Specifically, variant rs12144049 predicted independently associated with AD was suggested to affect the mRNA level of *CRNN* in the skin. The cornulin gene (*CRNN*) encodes a calcium-binding protein belonging to the "fused gene" family and may play a role in the mucosal/epithelial immune response and epidermal differentiation (http://www.genecards.org/, [66–68].

The *CRNN* gene was previously associated with AD (eczema) [66,67] as well as with the severe course of the disease, elevated IgE levels, eosinophilia, and concomitant asthma [67]. The *CRNN* gene is downregulated in the AD-like skin in the mouse model and human AD [66–68]. On the other hand, the GTExconsortium atlas data suggests that the AD risk allele of the *CRNN* gene (A of rs941934 [67]) is associated with the elevated *CRNN* expression in the healthy skin, and so is the disease risk variant C rs12144049 of the *FLG* gene (determined in the present study). One of the possible explanations of this inconsistency is that AD risk alleles indeed increase the *CRNN* gene expression in the healthy skin in some way, but this effect becomes opposite in the AD-like skin due to the significantly modified expression of the other cornified envelope proteins (*FLG*, *FLG2*, *LOR*, *CRNN*, *SPRR3v1*, *RPTN*, *HRNR*, *SPRR1Av1*) [68]. However, this assumption needs further experimental testing.

The present study determined no significant differences in the distribution of the *FLG* gene alleles and genotypes between male and female AD patients, which is in agreement with the previous report [69]. However, such differences were detected between affected and control females for allele C of rs12144049 within the additive and dominant genetic models. Several studies reported the higher susceptibility of females to AD [70–72]. The observed gender differences may be related to the influence of sex hormones (see, e.g., [73]).

Some limitations of the study should be acknowledged though. In particular, the male sample size was about two-fold smaller than that of females. This does not allow for making assumptions about a possible contribution of the *FLG* gene polymorphisms to AD in males.

## Conclusions

The results of the present study provide further support for the possible contribution of the *FLG* gene to AD in Caucasians from Central Russia. This contribution is apparently gender-specific and its exact mechanisms need clarifying.

## Supporting information

S1 Table The literature data about associations of the studied polymorphisms of the FLG genes (1q21.3) with AD (eczema) and some skin (psoriasis, ichthyosis vulgaris) and others allergic disorders (asthma, hay fever, etc.). (DOCX)

S2 Table The regulatory potential of the studied SNPs. (XLS)

S3 Table Gender-specific population parameters of the studied SNPs of the FLG gene in the AD and control groups. (DOCX)

S4 Table Regulatory effects of the AD-associated SNPs of the FLG gene and SNPs in high LD (r2≥0.80). (XLS)

S5 Table Non-synonymous SNPs in high LD (r2≥0.80) with the AD-associated locus rs3126085 of the FLG gene in females (HaploReg, v4.1, http://archive.broadinstitute.org/mammals/haploreg/haploreg.php). (DOCX)

S6 Table The eQTL effects of the AD-associated SNPs FLG gene in various tissues/organs. (XLS)

S7 Table eQTL values of AD-associated polymorphisms FLG gene in skin. (XLSX)

S8 Table eQTL values of SNPs in high LD (r2≥0.80) with the AD-associated polymorphisms FLG gene. (XLSX)

S9 Table eQTL values of SNPs in high LD (r2≥0.80) with the AD-associated polymorphisms FLG gene in skin. (XLSX)

S10 Table sQTL values of the studied SNPs of the FLG gene and SNPs linked to them (r2≥0.80). (XLSX)

S1 File References for S1 Table. (DOC)
