# Supplementary material for: Filaggrin gene polymorphisms are associated with atopic dermatitis in women but not in men in the Caucasian population of Central Russia
Source: PLoS One. 2021 Dec 9;16(12):e0261026. doi: 10.1371/journal.pone.0261026 (PMC8659355; doi:10.1371/journal.pone.0261026)
Supplement: S1 Table — (DOCX) [file pone.0261026.s001.docx]

Supplementary table 1

The literature data about associations of the studied polymorphisms of the *FLG* genes *(1q21.3)* with AD (eczema) and some skin (psoriasis, ichthyosis vulgaris) and others allergic disorders (asthma, hay fever, etc.)

| SNP | Position (hg38) | Фенотип | Association, significance | Reference |
| --- | --- | --- | --- | --- |
| rs12130219 | 152189630 | **AD** | **OR=0.66, р=1x10^-16^** | **Baurecht H.** **et al., 2015** |
|  |  | psoriasis | OR=1.15, р=4.0×10^−6^ | Baurecht H. et al., 2015 |
| rs558269137* (2282del4) | 152312601-152312604 | hay fever | OR**=**3.90, р<0.05 | [Schuttelaar M.L](https://www.ncbi.nlm.nih.gov/pubmed/?term=Schuttelaar%20ML%5BAuthor%5D&cauthor=true&cauthor_uid=19839980). et al., 2009 |
|  |  | eczema during the first year  of life | OR**=**8.20, р<0.05 | [Schuttelaar M.L](https://www.ncbi.nlm.nih.gov/pubmed/?term=Schuttelaar%20ML%5BAuthor%5D&cauthor=true&cauthor_uid=19839980). et al., 2009 |
|  |  | eczema up to 8 years | OR**=**6.00, р<0.05 | [Schuttelaar M.L](https://www.ncbi.nlm.nih.gov/pubmed/?term=Schuttelaar%20ML%5BAuthor%5D&cauthor=true&cauthor_uid=19839980). et al., 2009 |
|  |  | sensitization at the age of 8 years, which was enhanced by early-life cat exposure | OR**=**5.40, р<0.05 | [Schuttelaar M.L](https://www.ncbi.nlm.nih.gov/pubmed/?term=Schuttelaar%20ML%5BAuthor%5D&cauthor=true&cauthor_uid=19839980). et al., 2009 |
|  |  | hay fever from the age 5 years onwards | OR**=**3.90, р<0.05 | [Schuttelaar M.L](https://www.ncbi.nlm.nih.gov/pubmed/?term=Schuttelaar%20ML%5BAuthor%5D&cauthor=true&cauthor_uid=19839980). et al., 2009 |
|  |  | eczema in children | OR**=**0.67, р=5×10^-8^  combined wild type R501X and 2282del4 | [Henderson J](https://www.ncbi.nlm.nih.gov/pubmed/?term=Henderson%20J%5BAuthor%5D&cauthor=true&cauthor_uid=18325573). et al., 2008 |
|  |  | asthma in children | OR**=**1.80, р=1.9×10^-4^  combined R501X and 2282del4 | [Henderson J](https://www.ncbi.nlm.nih.gov/pubmed/?term=Henderson%20J%5BAuthor%5D&cauthor=true&cauthor_uid=18325573). et al., 2008 |
|  |  | asthma and eczema in children | OR**=**3.16, р=1.4×10^-11^  combined R501X and 2282del4 | [Henderson J](https://www.ncbi.nlm.nih.gov/pubmed/?term=Henderson%20J%5BAuthor%5D&cauthor=true&cauthor_uid=18325573). et al., 2008 |
|  |  | sensitization to multiple allergens | OR**=**2.12, р=5.42×10^-27^  combined R501X and 2282del4 | [Henderson J](https://www.ncbi.nlm.nih.gov/pubmed/?term=Henderson%20J%5BAuthor%5D&cauthor=true&cauthor_uid=18325573). et al., 2008 |
|  |  | eczema | OR**=**2.00, р<0.05  combined R501X, 2282del4 and R2447X | [Schuttelaar M.L](https://www.ncbi.nlm.nih.gov/pubmed/?term=Schuttelaar%20ML%5BAuthor%5D&cauthor=true&cauthor_uid=19839980). et al., 2009 |
|  |  | asthma | OR**=**3.70, р<0.05  combined R501X, 2282del4 and R2447X | [Schuttelaar M.L](https://www.ncbi.nlm.nih.gov/pubmed/?term=Schuttelaar%20ML%5BAuthor%5D&cauthor=true&cauthor_uid=19839980). et al., 2009 |
|  |  | AD | OR = 2.01, p = 0.007  combined R501X and 2282del4 | Ponińska J. et al., 2011 |
|  |  | allergic rhinitis | OR = 2.01, p = 0.007  combined R501X and 2282del4 | Ponińska J. et al., 2011 |
|  |  | atopic asthma | OR = 2.01, p = 0.007  combined R501X and 2282del4 | Ponińska J. et al., 2011 |
|  |  | eczema and asthma | RR=13.67, р<0.05  minor allele for at least one of the *FLG* variants R501X, 2282del4, or S3247X | Ziyab AH et al., 2014 |
|  |  | asthma and rhinitis | RR=7.46, р<0.05  minor allele for at least one of the *FLG* variants R501X, 2282del4, or S3247X | Ziyab AH et al., 2014 |
|  |  | eczema, asthma, and rhinitis | RR=23.44, р<0.05  minor allele for at least one of the *FLG* variants R501X, 2282del4, or S3247X | Ziyab AH et al., 2014 |
| rs61816761*  (R501X) | 152313385 | **allergic disease (asthma, hay fever and eczema)** | **OR=1.22, р=7x10^-21^** | **Ferreira M.A. et al., 2017** |
|  |  | **asthma** | **OR=1.26, р=5x10^-27^** | [**Zhu Z**](https://www.ncbi.nlm.nih.gov/pubmed/?term=Zhu%20Z%5BAuthor%5D&cauthor=true&cauthor_uid=31619474)**. et al., 2019** |
|  |  | **atopic asthma** | **р=1.5x10^-48^** | **Zhu Z. et al., 2019** |
|  |  | **early-onset asthma** | **р=4.1x10^-73^** | **Zhu Z. et al., 2019** |
|  |  | **asthma** | **OR=1.32, р=1x10^-30^** | **Ferreira MAR et al., 2019** |
|  |  | **asthma in children** | **OR=1.97, р=2x10^-65^** | [**Pividori M**](https://www.ncbi.nlm.nih.gov/pubmed/?term=Pividori%20M%5BAuthor%5D&cauthor=true&cauthor_uid=31036433)**. et al., 2019** |
|  |  | **asthma** | **β =-4.57, р=8x10^-27^** | [**Pividori M**](https://www.ncbi.nlm.nih.gov/pubmed/?term=Pividori%20M%5BAuthor%5D&cauthor=true&cauthor_uid=31036433)**. et al., 2019** |
|  |  | **severe asthma** | **OR=1.36, р=1x10^-8^** | **Shrine N. et al., 2019** |
|  |  | **eczema** | **р=8x10^-46^** | **Kichaev G. et al., 2019** |
|  |  | **asthma** | **OR=1.24, р=1x10^-22^** | **Olafsdottir TA et al., 2020** |
|  |  | ichthyosis vulgaris | р<0.05  combined R501X and 2282del4 | [Smith F.J](https://www.ncbi.nlm.nih.gov/pubmed/?term=Smith%20FJ%5BAuthor%5D&cauthor=true&cauthor_uid=16444271). et al., 2006 |
|  |  | AD | р<0.05  combined R501X and 2282del4 | [Weidinger S](https://www.ncbi.nlm.nih.gov/pubmed/?term=Weidinger%20S%5BAuthor%5D&cauthor=true&cauthor_uid=16815158). et al., 2006 |
|  |  | eczema in children | OR**=**0.67, р=5×10^-8^  combined wild type R501X and 2282del4 | [Henderson J](https://www.ncbi.nlm.nih.gov/pubmed/?term=Henderson%20J%5BAuthor%5D&cauthor=true&cauthor_uid=18325573). et al., 2008 |
|  |  | asthma in children | OR**=**1.80, р=1.9×10^-4^  combined R501X and 2282del4 | [Henderson J](https://www.ncbi.nlm.nih.gov/pubmed/?term=Henderson%20J%5BAuthor%5D&cauthor=true&cauthor_uid=18325573). et al., 2008 |
|  |  | asthma and eczema in children | OR**=**3.16, р=1.4×10^-11^  combined R501X and 2282del4 | [Henderson J](https://www.ncbi.nlm.nih.gov/pubmed/?term=Henderson%20J%5BAuthor%5D&cauthor=true&cauthor_uid=18325573). et al., 2008 |
|  |  | sensitization to multiple allergens | OR**=**2.12, р=5.42×10^-27^  combined R501X and 2282del4 | [Henderson J](https://www.ncbi.nlm.nih.gov/pubmed/?term=Henderson%20J%5BAuthor%5D&cauthor=true&cauthor_uid=18325573). et al., 2008 |
|  |  | eczema | OR**=**2.00, р<0.05  combined R501X, 2282del4 and R2447X | [Schuttelaar M.L](https://www.ncbi.nlm.nih.gov/pubmed/?term=Schuttelaar%20ML%5BAuthor%5D&cauthor=true&cauthor_uid=19839980). et al., 2009 |
|  |  | asthma | OR**=**3.70, р<0.05  combined R501X, 2282del4 and R2447X | [Schuttelaar M.L](https://www.ncbi.nlm.nih.gov/pubmed/?term=Schuttelaar%20ML%5BAuthor%5D&cauthor=true&cauthor_uid=19839980). et al., 2009 |
|  |  | AD | OR = 2.01, p = 0.007  combined R501X and 2282del4 | Ponińska J. et al., 2011 |
|  |  | allergic rhinitis | OR = 2.01, p = 0.007  combined R501X and 2282del4 | Ponińska J. et al., 2011 |
|  |  | atopic asthma | OR = 2.01, p = 0.007  combined R501X and 2282del4 | Ponińska J. et al., 2011 |
|  |  | eczema and asthma | RR=13.67, р<0.05  minor allele for at least one of the *FLG* variants R501X, 2282del4, or S3247X | Ziyab AH et al., 2014 |
|  |  | asthma and rhinitis | RR=7.46, р<0.05  minor allele for at least one of the *FLG* variants R501X, 2282del4, or S3247X | Ziyab AH et al., 2014 |
|  |  | eczema, asthma, and rhinitis | RR=23.44, р<0.05  minor allele for at least one of the *FLG* variants R501X, 2282del4, or S3247X | Ziyab AH et al., 2014 |
| rs3126085 | 152328341 | **AD** | **OR=0.82, p=5.90 × 10^−12^** | **Sun L. et al., 2011** |
|  |  | AD | OR=1.47, p=1.87 × 10^−8^ | [Schaarschmidt](javascript:void(0);) H. et al., 2015 |
|  |  | AD | р=1.11×10^–15^  combined rs3126085 *FLG* and rs17173197 *PRKAG2* | Shen С. et al.,2015 |
| rs12144049 | 152468434 | **AD** | **OR=1.53, p=3x10^-30^** | **Baurecht H.** **et al., 2015** |
|  |  | **AD** | **OR=1.39, p=1x10^-16^** | [**Schaarschmidt**](javascript:void(0);) **H.** **et al., 2015** |
|  |  | **atopic asthma** | **p=1.80×10^-21^** | **Zhu Z. et al., 2019** |
|  |  | **early-onset asthma** | **p=2.30×10^-27^** | **Zhu Z. et al., 2019** |
| rs6661961 | 152469813 | **AD** | **β=0.34, р=9x10^-11^** | **Weidinger S.** **et al., 2013** |
| rs471144 | 152481779 | **AD** | **OR=1.54, p=2x10^-12^** | **Baurecht H.** **et al., 2015** |
| rs10888499 | 152560266 | **AD** | **OR=1.49, р=5x10^-25^** | **Baurecht H.** **et al., 2015** |
| rs77199844 (del) | 152784619-152784620 | **AD** | **OR=1.23, р=2x10^-17^** | **Baurecht H.** **et al., 2015** |
| rs4363385 | 153016845 | **AD** | **OR=1.23, p=2x10^-17^** | **Baurecht H. et al., 2015** |
|  |  | **psoriasis** | **OR=0.89, р<10^−8^** | **Baurecht H. et al., 2015** |

Note: * loss-of-function variants of the *FLG* gene; GWAS data are shown in bold

.
