# Supplementary material for: Filaggrin gene polymorphisms are associated with atopic dermatitis in women but not in men in the Caucasian population of Central Russia
Source: PLoS One. 2021 Dec 9;16(12):e0261026. doi: 10.1371/journal.pone.0261026 (PMC8659355; doi:10.1371/journal.pone.0261026)
Supplement: S3 Table — (DOCX) [file pone.0261026.s003.docx]

Supplementary table 3

Gender-specific population parameters of the studied SNPs of the *FLG* gene in the AD and control groups

|  | SNP | Minor allele | Major allele | Minor allele frequency | Number of the studied chromosomes | Genotype distribution* | H_o_ | H_e_ | Р_HWE_ |
| --- | --- | --- | --- | --- | --- | --- | --- | --- | --- |
| Females | AD patients (n=474) | | | | | | | | |
|  | rs12130219 | G | A | 0.226 | 924 | 22/165/275 | 0.357 | 0.350 | 1.000 |
|  | rs558269137 | delACTG | ACTG | 0.032 | 932 | 0/30/436 | 0.064 | 0.062 | 1.000 |
|  | rs6661961 | T | A | 0.409 | 936 | 91/201/176 | 0.429 | 0.484 | 0.079 |
|  | rs3126085 | A | G | 0.148 | 924 | 16/105/341 | 0.227 | 0.253 | 0.188 |
|  | rs12144049 | C | T | 0.228 | 916 | 18/173/267 | 0.378 | 0.352 | 0.350 |
|  | rs61816761 | A | G | 0.011 | 940 | 0/10/460 | 0.021 | 0.021 | 1.000 |
|  | rs471144 | G | T | 0.084 | 928 | 8/62/394 | 0.134 | 0.154 | 0.062 |
|  | rs10888499 | C | A | 0.249 | 936 | 36/161/271 | 0.344 | 0.374 | 0.219 |
|  | rs77199844 | delAT | AT | 0.052 | 932 | 0/48/418 | 0.103 | 0.098 | 1.000 |
|  | rs4363385 | T | C | 0.426 | 904 | 91/203/158 | 0.449 | 0.489 | 0.276 |
|  | Сontrol group (n=432) | | | | | | | | |
|  | rs12130219 | G | A | 0.234 | 860 | 32/137/261 | 0.319 | 0.358 | 0.124 |
|  | rs558269137 | delACTG | ACTG | 0.016 | 852 | 0/14/412 | 0.033 | 0.032 | 1.000 |
|  | rs6661961 | T | A | 0.415 | 856 | 79/197/152 | 0.460 | 0.486 | 0.484 |
|  | rs3126085 | A | G | 0.095 | 844 | 8/64/350 | 0.152 | 0.172 | 0.097 |
|  | rs12144049 | C | T | 0.152 | 808 | 6/111/287 | 0.275 | 0.258 | 0.582 |
|  | rs61816761 | A | G | 0.002 | 856 | 0/2/426 | 0.005 | 0.005 | 1.000 |
|  | rs471144 | G | T | 0.069 | 816 | 0/56/352 | 0.137 | 0.128 | 0.605 |
|  | rs10888499 | C | A | 0.282 | 856 | 20/201/207 | 0.470 | 0.405 | 0.027 |
|  | rs77199844 | delAT | AT | 0.062 | 836 | 0/52/366 | 0.124 | 0.117 | 1.000 |
|  | rs4363385 | T | C | 0.439 | 840 | 77/215/128 | 0.512 | 0.493 | 0.576 |

Supplementary table 3 (continued)

The allele and genotype frequencies of the studied *FLG* gene SNPs in the AD and control groups of female and male

|  | SNP | Minor allele | Major allele | Minor allele frequency | Number of the studied chromosomes | Genotype distribution* | H_o_ | H_e_ | Р_HWE_ |
| --- | --- | --- | --- | --- | --- | --- | --- | --- | --- |
| Males | AD patients (n=226) | | | | | | | | |
|  | rs12130219 | G | A | 0.277 | 444 | 12/99/111 | 0.446 | 0.401 | 0.343 |
|  | rs558269137 | delACTG | ACTG | 0.023 | 440 | 0/10/210 | 0.045 | 0.044 | 1.000 |
|  | rs6661961 | T | A | 0.391 | 448 | 33/109/82 | 0.487 | 0.476 | 0.843 |
|  | rs3126085 | A | G | 0.142 | 444 | 6/51/165 | 0.230 | 0.244 | 0.439 |
|  | rs12144049 | C | T | 0.207 | 440 | 8/75/137 | 0.341 | 0.328 | 1.000 |
|  | rs61816761 | A | G | 0.004 | 448 | 0/2/222 | 0.009 | 0.009 | 1.000 |
|  | rs471144 | G | T | 0.049 | 452 | 0/22/204 | 0.097 | 0.093 | 1.000 |
|  | rs10888499 | C | A | 0.246 | 452 | 16/79/131 | 0.350 | 0.371 | 0.615 |
|  | rs77199844 | delAT | AT | 0.080 | 448 | 0/36/188 | 0.161 | 0.148 | 1.000 |
|  | rs4363385 | T | C | 0.417 | 448 | 43/101/80 | 0.451 | 0.486 | 0.560 |
|  | Сontrol group (n=180) | | | | | | | | |
|  | rs12130219 | G | A | 0.224 | 352 | 10/59/107 | 0.335 | 0.348 | 0.756 |
|  | rs558269137 | delACTG | ACTG | 0.030 | 336 | 0/10/158 | 0.060 | 0.058 | 1.000 |
|  | rs6661961 | T | A | 0.371 | 348 | 33/63/78 | 0.362 | 0.466 | 0.064 |
|  | rs3126085 | A | G | 0.075 | 348 | 0/26/148 | 0.149 | 0.138 | 1.000 |
|  | rs12144049 | C | T | 0.194 | 324 | 12/39/111 | 0.241 | 0.313 | 0.035 |
|  | rs61816761 | A | G | 0.000 | 344 | 0/0/172 | 0.000 | 0.000 | 1.000 |
|  | rs471144 | G | T | 0.073 | 356 | 2/22/154 | 0.124 | 0.135 | 0.376 |
|  | rs10888499 | C | A | 0.299 | 344 | 16/71/85 | 0.413 | 0.420 | 0.799 |
|  | rs77199844 | delAT | AT | 0.056 | 338 | 0/20/158 | 0.112 | 0.106 | 1.000 |
|  | rs4363385 | T | C | 0.408 | 336 | 31/75/62 | 0.446 | 0.483 | 0.550 |

Note: * minor allele homozygotes / heterozygotes / major allele homozygotes
