# Supplementary material for: Filaggrin gene polymorphisms are associated with atopic dermatitis in women but not in men in the Caucasian population of Central Russia
Source: PLoS One. 2021 Dec 9;16(12):e0261026. doi: 10.1371/journal.pone.0261026 (PMC8659355; doi:10.1371/journal.pone.0261026)
Supplement: S5 Table — (DOCX) [file pone.0261026.s005.docx]

Supplementary table 5

Non-synonymous SNPs in high LD (r^2^≥0.80) with the AD-associated locus rs3126085 of the *FLG* gene in females (HaploReg, v4.1, <http://archive.broadinstitute.org/mammals/haploreg/haploreg.php>)

| nsSNPs | Position (hg38) | r² | D' | Amino acid change | *eEnsembl* database (<http://www.ensembl.org/index.html>) | | | |
| --- | --- | --- | --- | --- | --- | --- | --- | --- |
|  |  |  |  |  | SIFT database | | PolyPhen-2 database | |
|  |  |  |  |  | Score | Predicted effects | Score | Predicted effects |
| rs77422831 | 152303673 | 0.97 | 0.99 | Arg3738Leu | 0.12 | «tolerated» | **0.836** | **«possibly damaging»** |
| rs72697000 | 152304296 | 0.96 | 0.98 | Arg3530Ser | 0.73 | «tolerated» | **0.998** | **«possibly damaging»** |
| rs3126067 | 152304413 | 0.95 | 1 | Asn3491Lys | 0.43 | «tolerated» | **0.766** | **«possibly damaging»** |
| rs2065955 | 152304579 | 0.95 | 0.99 | Gly3436Ala | 0.08 | «tolerated» | **0.748** | **«possibly damaging»** |
| rs3126072 | 152307253 | 0.81 | 1 | Gly2545Arg | 0.45 | «tolerated» | 0.000 | «benign» |
| rs3126074 | 152307365 | 0.82 | 1 | His2507Gln | 1.00 | «tolerated» | 0.000 | «benign» |
| rs55650366 | 152307444 | 0.81 | 0.99 | Leu2481Trp | 0.11 | «tolerated» | 0.315 | «benign» |
| rs7522925 | 152308563 | 0.86 | 1 | Ala2108Val | 0.06 | «tolerated» | 0.021 | «benign» |
| rs3126079 | 152309003 | 0.8 | 1 | His1961Gln | 0.81 | «tolerated» | 0.021 | «benign» |
| rs12407748 | 152309214 | 0.98 | 1 | Arg1891Pro | **0.03** | **«deleterious»** | **0.954** | **«probably damaging»** |
| rs12405241 | 152309472 | 0.98 | 0.99 | Ala1805Val | 0.20 | «tolerated» | **0.959** | **«probably damaging»** |
| rs12405278 | 152309791 | 0.99 | 1 | Arg1699Cys | **0.02** | **«deleterious»** | **0.912** | **«probably damaging»** |
| rs12407807 | 152309835 | 0.99 | 1 | Arg1684Pro | 0.05 | «tolerated» | **0.978** | **«probably damaging»** |
| rs11204978 | 152310441 | 0.98 | 0.99 | Ser1482Phe | **0.00** | **«deleterious»** | **0.841** | **«probably damaging»** |
| rs11586631 | 152310807 | 0.82 | 1 | Arg1360Leu | 0.08 | «tolerated» | 0.136 | «benign» |
| rs3120653 | 152312378 | 0.8 | 0.99 | Asp836Glu | 1.00 | «tolerated» | 0.000 | «benign» |
| rs74129461 | 152312623 | 0.8 | 0.98 | Glu755Lys | **0.03** | **«deleterious»** | 0.224 | «benign» |
| rs11584340 | 152313454 | 0.82 | 1 | Pro478Ser | 1.00 | «tolerated» | 0.034 | «benign» |
| rs2011331 | 152313526 | 0.81 | 1 | Thr454Ala | 1.00 | «tolerated» | 0.006 | «benign» |
| rs11588170 | 152313556 | 0.99 | 1 | Glu444Arg | 0.18 | «tolerated» | **0.816** | **«possibly damaging»** |
| rs41267154 | 152313891 | 0.82 | 1 | Glu332Val | **0.04** | **«deleterious»** | **0.995** | **«probably damaging»** |
