# Supplementary material for: Filaggrin gene polymorphisms are associated with atopic dermatitis in women but not in men in the Caucasian population of Central Russia
Source: PLoS One. 2021 Dec 9;16(12):e0261026. doi: 10.1371/journal.pone.0261026 (PMC8659355; doi:10.1371/journal.pone.0261026)
Supplement: S1 File — (DOC) [file pone.0261026.s011.doc]

**References**

1. Baurecht H, Hotze M, Brand S, et al. Genome-wide comparative analysis of atopic dermatitis and psoriasis gives insight into opposing genetic mechanisms. *Am J Hum Genet*. 2015;96(1):104–120. doi:10.1016/j.ajhg.2014.12.004
2. Ferreira M.A., Vonk J.M., Baurecht H., et al. Shared genetic origin of asthma, hay fever and eczema elucidates allergic disease biology. Nat Genet. 2017;49(12):1752–1757.
3. Ferreira M.A.R., Mathur R., Vonk J.M., et al. Genetic Architectures of Childhood- and Adult-Onset Asthma Are Partly Distinct. Hum Genet. 2019;104(4):665–684.
4. Henderson J., Northstone K., Lee S.P., et al. The burden of disease associated with filaggrin mutations: a population-based, longitudinal birth cohort study. Allergy Clin Immunol. 2008;121(4):872-877.
5. Kichaev G., Bhatia G., Loh P.R., et al. Leveraging Polygenic Functional Enrichment to Improve GWAS Power. Am J Hum Genet. 2019;104(1):65–75.
6. Olafsdottir TA, Theodors F, Bjarnadottir K, Bjornsdottir US, Agustsdottir AB, Stefansson OA, Ivarsdottir EV, Sigurdsson JK, Benonisdottir S, Eyjolfsson GI, Gislason D, Gislason T, Guðmundsdóttir S, Gylfason A, Halldorsson BV, Halldorsson GH, Juliusdottir T, Kristinsdottir AM, Ludviksdottir D, Ludviksson BR, Masson G, Norland K, Onundarson PT, Olafsson I, Sigurdardottir O, Stefansdottir L, Sveinbjornsson G, Tragante V, Gudbjartsson DF, Thorleifsson G, Sulem P, Thorsteinsdottir U, Norddahl GL, Jonsdottir I, Stefansson K. Eighty-eight variants highlight the role of T cell regulation and airway remodeling in asthma pathogenesis. Nat Commun. 2020 Jan 20;11(1):393. doi: 10.1038/s41467-019-14144-8.
7. Pividori M., Schoettler N., Nicolae D.L., et al. Shared and distinct genetic risk factors for childhood-onset and adult-onset asthma: genome-wide and transcriptome-wide studies. Lancet Respir Med. 2019;7(6):509-522.
8. Ponińska J, Samoliński B, Tomaszewska A, et al. Filaggrin gene defects are independent risk factors for atopic asthma in a Polish population: a study in ECAP cohort. *PLoS One*. 2011;6(2):e16933.
9. Schaarschmidt H., Ellinghaus D., Rodríguez E., et al. A genome-wide association study reveals 2 new susceptibility loci for atopic dermatitis. Allergy and Clinical Immunology. 2015;136(3):802 – 806.
10. Schuttelaar M.L., Kerkhof M., Jonkman M.F., et al. Filaggrin mutations in the onset of eczema, sensitization, asthma, hay fever and the interaction with cat exposure. Allergy. 2009;64(12):1758-65.
11. Shen С., Liu L.,·Jiang Z.,·et al. Four genetic variants interact to confer susceptibility to atopic dermatitis in Chinese Han population. Molecular Genetics and Genomics. 2015;290(4): 1493-1498.
12. Shrine N., Portelli M.A., John C., et al. Moderate-to-severe asthma in individuals of European ancestry: a genome-wide association study. Lancet Respir Med. 2019;7(1):20–34.
13. Smith F.J., Irvine A.D., Terron-Kwiatkowski A., et al. Loss-of-function mutations in the gene encoding filaggrin cause ichthyosis vulgaris. Nat Genet. 2006;38(3):337-342.
14. Sun L.D., Xiao F.L., Li Y., et al. Genome-wide association study identifies two new susceptibility loci for atopic dermatitis in the Chinese Han population. Nat Genet. 2011;43:690–694.
15. Weidinger S., Illig T., Baurecht H., et al. Loss-of-function variations within the filaggrin gene predispose for atopic dermatitis with allergic sensitizations. Allergy Clin Immunol. 2006;118(1):214-219.
16. Weidinger S., Willis-Owen S.A., Kamatani Y., et al. A genome-wide association study of atopic dermatitis identifies loci with overlapipng effects on asthma and psoriasis. Hum Mol Genet. 2013;22(23):4841–4856.
17. Zhu Z., Zhu X., Liu C.L., et al. Shared genetics of asthma and mental health disorders: a large-scale genome-wide cross-trait analysis. Eur Respir J. 2019;54(6): 1901507.
18. Ziyab AH, Karmaus W, Zhang H, Holloway JW, Steck SE, Ewart S, Arshad SH. Allergic sensitization and filaggrin variants predispose to the comorbidity of eczema, asthma, and rhinitis: results from the Isle of Wight birth cohort. Clin Exp Allergy. 2014 Sep;44(9):1170-8. doi: 10.1111/cea.12321.
